# Methodological Modifications of the INFOGEST 2.0 Protocol in Plant‐Based Food Studies: A Systematic Review

**DOI:** 10.1111/1750-3841.71356

**Published:** 2026-08-21

**Authors:** Matheus Braga Mendes, Giovana Gomes Garcia Rodrigues, Maria Esther Leal da Silva Sad, Giovanna Ferreira Paiva dos Santos, Juliana de Carvalho da Costa

**Affiliations:** ^1^ Department of Pharmaceutical Sciences Federal University of Juiz de Fora Juiz de Fora Minas Gerais Brazil

## Abstract

The INFOGEST 2.0 protocol is widely applied as a standardized static in vitro digestion model, and its use in studies involving plant‐based foods has increased substantially in recent years. This systematic review aimed to identify, classify, and critically evaluate methodological modifications applied to the INFOGEST 2.0 protocol in studies investigating plant‐based foods and supplements. A systematic search was conducted in the ScienceDirect, Scopus, and PubMed database, identifying 119 original research articles published between 2019 and 2024. Methodological modifications across the oral, gastric, and intestinal phases were extracted and analyzed according to the parameters altered and their underlying rationale. Approximately, half of studies (50.4%) deviated from standardized INFOGEST conditions, particularly regarding enzyme activity, pH, bile concentration, and digestion time. Importantly, these adaptations were not exclusively driven by the plant‐based nature of the food matrices, but also reflected methodological factors related to the study population or objectives, including aging‐related physiological conditions, specific experimental needs, the tracing of particular compounds (e.g., minerals and phenolic compounds), and blank subtraction. While such modifications may enhance physiological relevance or analytical accuracy, they introduce a trade‐off with interstudy comparability, highlighting the need for clearer reporting practices and consensus‐based extensions of the INFOGEST framework to balance physiological relevance with methodological consistency.

## 1 Introduction

The INFOGEST protocol is an internationally harmonized static in vitro digestion method designed to simulate human gastrointestinal conditions in a physiologically relevant and reproducible manner. First published in 2014 through an international scientific consensus, the protocol was developed to harmonize in vitro digestion procedures and improve reproducibility in intra‐ and interlaboratory studies (Minekus et al. 2014). It standardizes key experimental parameters in each digestive phase, including enzyme activities, pH values, electrolyte composition, and digestion times, thereby enabling greater consistency and comparability among studies investigating nutrient digestibility. In 2019, the revised INFOGEST 2.0 protocol was published to refine the original method by incorporating updates such as the oral phase, gastric lipase, and recommendations for optimal reagents to better mimic human digestion (Brodkorb et al. 2019).

The importance of INFOGEST continues to grow alongside the rapid expansion of the plant‐based food and supplement sector. Driven by nutritional, environmental, and consumer‐health motivations, plant‐based products have diversified across categories such as meat analogs, beverages, flours, protein isolates, and fortified supplements (Ferrara et al. 2025).

Market analyses indicate sustained growth in both global and regional demand, reinforcing the need for robust and standardized analytical tools capable of evaluating the nutritional performance of these increasingly complex matrices. The global plant‐based market is projected to reach substantially higher values by 2030 or 2031, with some reports estimating it could exceed USD 100 billion, driven by a strong compound annual growth rate (CAGR), particularly in emerging regions (The Good Food Institute 2023).

However, comparing digestion results across plant‐based studies remains challenging. This difficulty is partly related to the structural and biochemical complexity of plant matrices, including factors such as fiber content, cell wall integrity, protein aggregation, and antinutritional compounds, which can affect protein hydrolysis and nutrient release (Joye 2019; Lucas‐González et al. 2023). In addition, differences in experimental objectives, target populations, and methodological choices may lead researchers to adapt specific INFOGEST 2.0 conditions, further affecting the comparability of results.

These specific challenges may require careful consideration when applying standardized in vitro digestion methods to plant‐based foods. In this context, some researchers have applied INFOGEST 2.0 with specific modifications when studying plant‐based matrices (Martineau‐Côté et al. 2023). Such adaptations, together with other methodological deviations from the standardized protocol, may contribute to heterogeneity across the literature and affect the comparability of results (Opazo‐Navarrete et al. 2025).

Although the number of plant‐based studies using INFOGEST 2.0 has increased substantially, no review to date has systematically mapped how these studies modify the protocol, whether these modifications are justified and how they may affect physiological relevance or reproducibility. This methodological fragmentation limits the interpretability and comparability of findings in the field.

Therefore, the objective of this systematic review is to identify, categorize and critically analyze the methodological modifications applied to the static INFOGEST 2.0 protocol in studies evaluating plant‐based foods and supplements, and to interpret the motivations behind these deviations and their implications for physiological relevance and reproducibility.

## 2 Material and Methods

**FIGURE 1:**
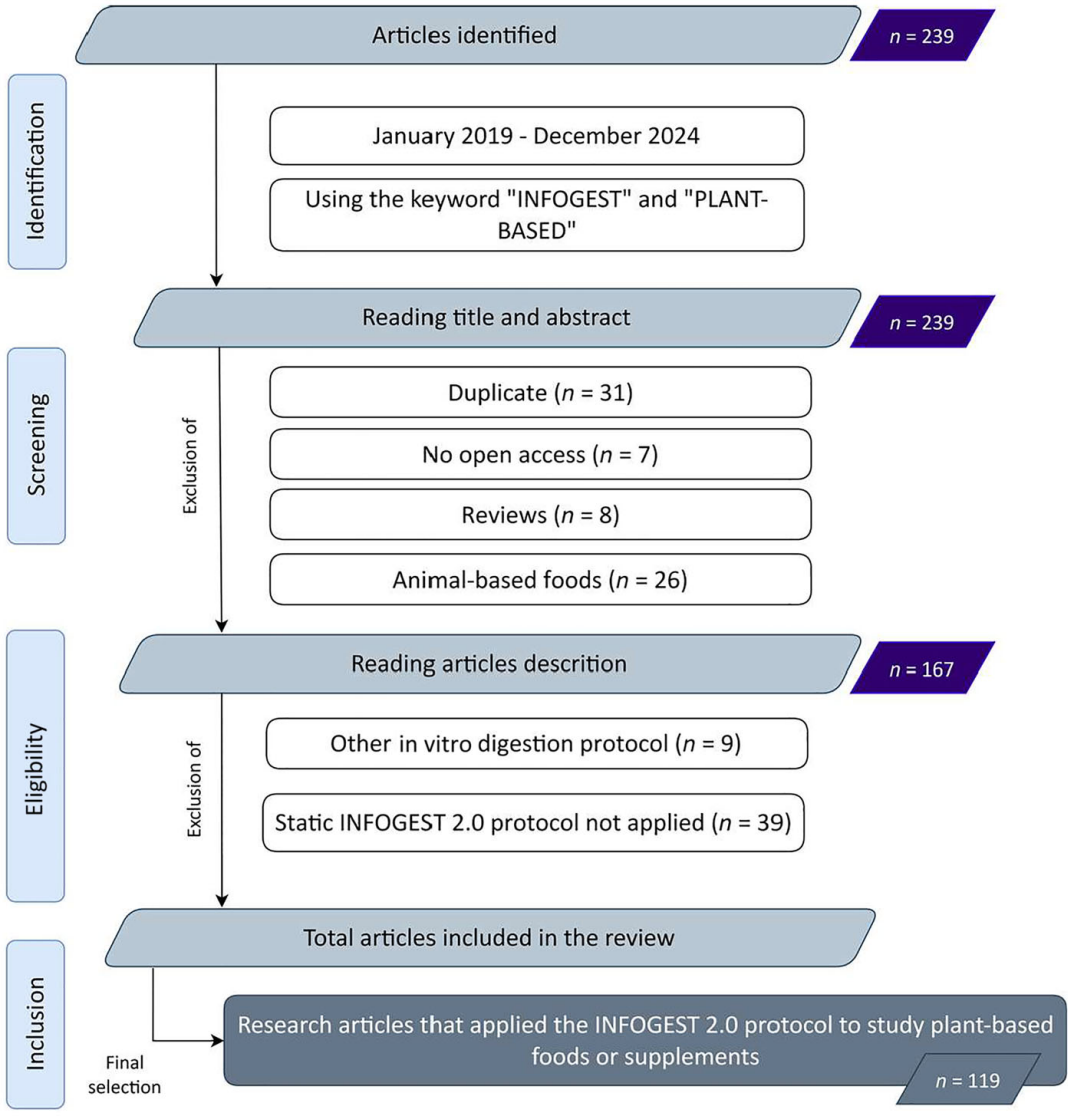
PRISMA diagram of the literature search and study selection process.

A systematic search was conducted on ScienceDirect, Scopus, and PubMed employing the keywords “INFOGEST” and “PLANT‐BASED” to identify relevant articles. The research was applied in all document sections and was limited to original research articles from 2019 to 2024, identifying 239 articles. The screening process began with the evaluation of titles and abstracts. At this stage, duplicate articles (*n* = 31), articles without open access (*n* = 7), review articles (*n* = 8), and studies related to animal‐based foods (*n* = 26) were excluded. The remaining articles were then assessed through full‐text reading. During this stage, studies that used in vitro digestion protocols other than INFOGEST (*n* = 9) and those that did not follow the static INFOGEST 2.0 protocol, such as semidynamic or dynamic (*n* = 39), were excluded. After applying these criteria, 119 research articles that used the static INFOGEST 2.0 protocol to investigate the digestion of plant‐based foods or supplements were included in this review. The manuscript retrieval and selection process, including the application of inclusion and exclusion criteria, was conducted according to the PRISMA approach and is summarized in Figure 1. The analyzed studies were classified according to the number of methodological modifications applied to the digestion protocol. For clarity, the studies were grouped into four categories (one, two, three, and four or more modifications), as presented in Supporting Information Tables I–IV.

## 3 Results and Discussion

### 3.1 Main Modifications

The INFOGEST protocol emerged as a standardized in vitro digestion method to facilitate the comparison of results across different studies, as previously mentioned. For this reason, researchers strongly recommend applying the protocol under the same experimental conditions described in the original publication to ensure reproducibility. Since the publication of its first version in 2014, the INFOGEST protocol has undergone continuous revisions, culminating in the release of the second version in 2019 (INFOGEST 2.0). However, the present review covers studies published between 2019 and 2024, a period during which specific standardized adaptations for certain physiological conditions were still emerging. For example, several studies included in this review introduced modifications to simulate digestion in older adults before the publication of the age‐adapted INFOGEST protocol proposed by Ménard, Lesmes, et al. (2023), which standardized conditions to represent adults over 65 years of age. Therefore, some of the modifications identified in studies involving older adults may reflect the absence, at the time, of a specific consensus protocol for this population. Following the publication of Ménard, Lesmes, et al. (2023), future studies are expected to adopt this age‐adapted protocol more consistently, improving comparability among studies focused on older adults.

More recently, Sousa et al. (2023a) and Egger et al. (2026) expanded the static INFOGEST 2.0 protocol by standardizing an in vitro method for determining protein digestibility and the Digestible Indispensable Amino Acid Score (DIAAS) of dietary proteins, considering both the soluble fraction (supernatant) and the insoluble fraction (pellet).

In the present review, approximately half of the studies analyzed (*n* = 59) strictly followed the static INFOGEST 2.0 protocol, whereas the remaining studies (*n* = 60) introduced modifications in one or more digestion phases, including the oral, gastric, and intestinal phases. These adaptations were associated with different methodological rationales, particularly the need to simulate specific physiological conditions or to address targeted experimental objectives. Although some modifications were related to the structural and compositional characteristics of plant‐based food matrices, most were implemented to better represent particular digestion scenarios, especially those involving older adults, reflecting the growing interest in age‐related nutritional challenges.

Taken together, the modifications identified across the oral, gastric, and intestinal phases demonstrate a high degree of methodological variability among studies applying the *static* INFOGEST 2.0 protocol to plant‐based foods. While such adaptations may be justified by the specific characteristics of the food matrix, target population, or research objective, they may also limit the direct comparison and reproducibility of digestion outcomes across the literature. This is particularly relevant because the main purpose of the INFOGEST protocol is to harmonize in vitro digestion procedures and improve intra‐ and interstudy comparability.

**FIGURE 2:**
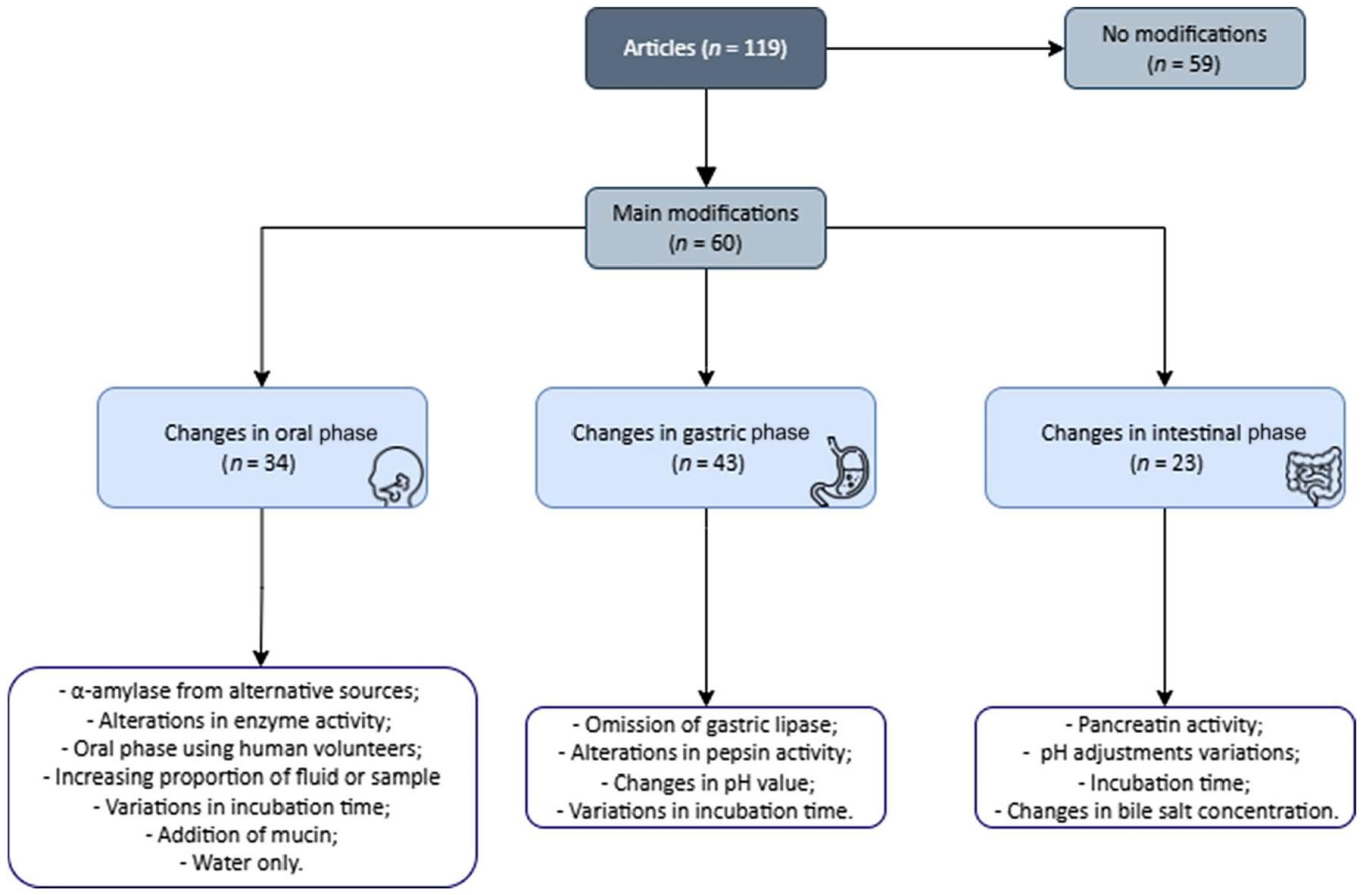
Distribution of methodological modifications reported in studies applying the INFOGEST in vitro digestion protocol according to the digestion phase. * The sum of the total modifications was higher than the number of articles analyzed, due to the fact that some articles presented more than one modification.

Therefore, the methodological adaptations identified in this review were categorized according to the digestion phase in which they occurred: oral, gastric, or intestinal. The frequency and type of these modifications are summarized in Figure 2, highlighting the predominance of changes in the oral and gastric phases.

#### 3.1.1 Oral Phase

Physiologically, the digestion process begins in the mouth with the secretion of saliva containing the enzyme α‐amylase (EC 3.2.1.1). This enzyme catalyzes the hydrolysis of internal α‐1,4‐glycosidic linkages in polysaccharides, producing D‐glucose, maltose, oligosaccharides, and dextrins (Peyrot des Gachons and Breslin 2016). To closely mimic the physiological conditions of the oral cavity, the INFOGEST protocol establishes specific parameters to be followed.

**FIGURE 3:**
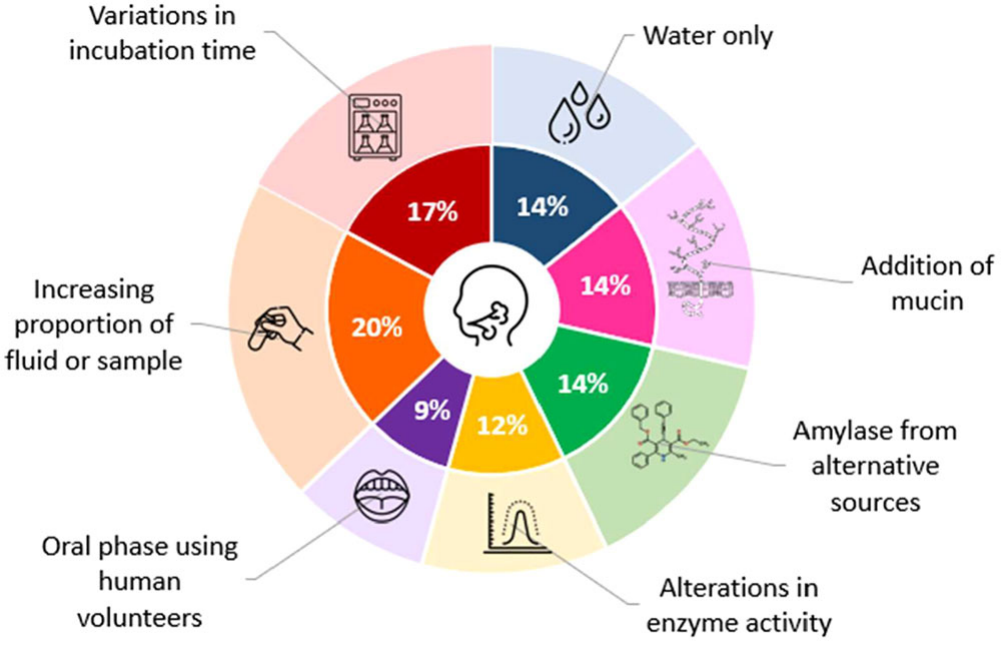
Distribution of methodological modifications reported in the oral phase (*n* = 34).

The main modifications observed in the oral phase include use of α‐amylase from alternative sources (*n* = 5), alterations in enzyme activity (*n* = 4), performing the oral phase using human volunteers (*n* = 4), water only (*n* = 5), addition of mucin (*n* = 5), increasing proportion of fluid or sample (*n* = 7), and variations of incubation time (*n* = 6), as shown in Figure 3 and presented in Supporting Information Tables I–IV. The INFOGEST protocol allows the optional inclusion of α‐amylase, as recommended when starch‐containing foods are being digested (Brodkorb et al. 2019).

Another common modification involves the use of α‐amylase from alternative sources. Among the studies analyzed, microbial α‐amylase, particularly from *Bacillus* species, was the most used alternative (Muleya, Young, et al. 2023; Muleya, Li, et al. 2023; Muleya et al. 2024). These sources substitutions are not recommended due to biochemical differences between amylases catalytic modes: human salivary α‐amylase functions act as an endoamylase, hydrolyzing internal glycosidic bonds within polysaccharides, whereas microbial α‐amylases often act as exoamylases, cleaving glycosidic bonds from the nonreducing ends (Gupta et al. 2003).

These differences can impact the digestion profile, particularly when studying carbohydrate digestion, and compromise the comparability and interpretation of results across studies.

Variations in enzyme activity can significantly influence the digestion process during the oral phase. Adjustments in enzyme concentrations have been adopted in some studies to better mimic specific physiological conditions, particularly those associated with the elderly population, in which age‐related functional decline affects digestive processes (Sousa et al. 2023a). Increased salivary α‐amylase activity has been reported as an attempt to reproduce changes observed in older adults, which may subsequently affect the accessibility of proteases and lipases to their substrates during the gastric and intestinal phases (Melchior et al. 2023). However, the protocol proposed by Ménard, Lesmes, et al. (2023) within the INFOGEST network recommends maintaining salivary α‐amylase activity at 75 U/mL, consistent with the value established for the adult model.

Another notable modification adopted by some authors is the performance of the oral phase using human volunteers (Ariz et al. 2024; Bocchi et al. 2021; Zahir et al. 2021). Given that solid food samples must be broken down into small particles through simulated mastication and the protocol recommends the use of human salivary α‐amylase, conducting the oral phase in vivo may seem like an attractive approach. However, in these studies, enzymatic activity is typically not directly measured or standardized, which introduces an additional source of variability. Salivary α‐amylase levels can vary substantially between individuals and even within the same individual depending on physiological conditions, making it difficult to control or reproduce digestion conditions.

Furthermore, several factors can influence oral food processing, including chewing time, dental condition, bite force, and other individual differences. The simulated oral phase in the INFOGEST protocol is standardized to 2 min. Performing it for shorter or uncontrolled durations, as often occurs in vivo, may compromise both the accuracy and reproducibility of results under laboratory conditions (Minekus et al. 2014).

#### 3.1.2 Gastric Phase

After the oral phase, the bolus is swallowed, and the digestive process continues in the stomach. The simulated gastric phase involves the addition of simulated gastric fluid (SGF), digestive enzymes (pepsin and gastric lipase), and a specific acidic environment to replicate physiological conditions essential for optimal enzymatic activity.

**FIGURE 4:**
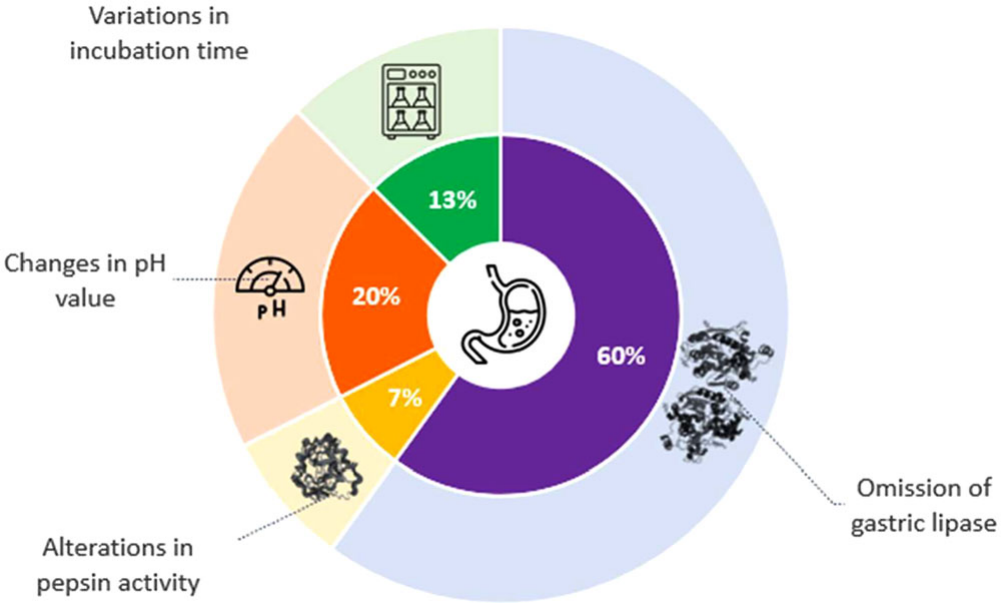
Distribution of methodological modifications reported in the gastric phase (*n* = 43).

The main modifications observed in the gastric phase included the omission of gastric lipase (*n* = 24), alterations in pepsin activity (*n* = 3), changes in pH value (*n* = 8), and variations in incubation time (*n* = 5), as shown in Figure 4 and presented in Supporting Information Tables I–IV. According to the revised INFOGEST protocol, the inclusion of gastric lipase is recommended, as lipid digestion begins in the stomach. This enzyme enhances the effectiveness of pancreatic lipase by initiating the breakdown of lipid substrates that may otherwise be poorly digested by pancreatic lipase alone (Brodkorb et al. 2019).

However, despite this recommendation, several studies have omitted gastric lipase from the digestion procedure, which may be explained by practical limitations, such as the limited availability of commercially accessible gastric lipase or suitable alternatives, and by the specific focus of the studies. In investigations primarily targeting proteins, carbohydrates, peptides, or phenolic compounds, gastric lipase may be considered less critical, since pepsin and pancreatin are generally the main enzymes involved in the digestion and release of these compounds (Brodkorb et al. 2019; Van de Walle et al. 2024; Purwandari et al. 2023; Moreira et al. 2024; Cañas et al. 2024).

Another modification observed in some studies was the reduction in pepsin activity, with reported values ranging from 400 to 1500 U/mL (Abdelbost et al. 2024; Melchior et al. 2023; Song et al. 2024). These adjustments are often proposed to better represent gastric digestion in specific populations, particularly older adults, for whom age‐related physiological changes may affect digestive function (Melchior et al. 2023; Song et al. 2024). In this context, lower pepsin activities have been employed to reproduce the decreased gastric secretory capacity commonly observed with aging, with some studies reporting reductions of up to fourfold (Wang, Rousta, et al. 2024). These modifications may significantly affect proteolytic efficiency and digestion kinetics. Supporting this, experimental findings demonstrated that elderly‐adapted digestion conditions reduced the digestibility of whey and rice proteins by approximately 20%, and pea protein by around 10%, suggesting that standardized protocols may overestimate nutrient bioaccessibility and absorption in vulnerable populations (Melchior et al. 2023).

Modifications to the pH of the gastric phase have also been documented and can significantly influence digestion outcomes (Abdelbost et al. 2024; De Paiva Gouvêa et al. 2024; Gutiérrez‐Luna et al. 2023; Kurapkienė et al. 2024; Melchior et al. 2023; Moretton et al. 2023; Wang, Chang, et al. 2024). In vivo, gastric pH is dynamic and influenced by factors such as meal composition, age, health conditions, and medication use (Patel et al. 2020; Ménard, Lesmes, et al. 2023). Nevertheless, the INFOGEST protocol recommends maintaining a constant pH during the gastric phase to ensure optimal enzyme activity and reliable digestion outcomes.

Incubation time was also modified in some studies, with durations ranging from 1 to 3 h (Adiamo et al. 2023; Maldonado et al. 2023; Melchior et al. 2023; Moretton et al. 2023). The standardized protocol suggests a 2 h incubation for the adult gastric phase. However, some authors increased the incubation time to better reflect gastric emptying in older adults (Melchior et al. 2023; Moretton et al. 2023), as gastric transit tends to be slower in this population (Ménard, Lesmes, et al. 2023). These variations can impact the in vitro digestion process, as the contact time between the oral bolus and gastric enzymes must be sufficient to allow effective enzymatic action.

#### 3.1.3 Intestinal Phase

The digestive process continues with the intestinal phase, during which the acidic gastric chyme is neutralized to match the physiological pH of the small intestine. It is important to highlight that the INFOGEST protocol simulates only the upper portion of the human gastrointestinal tract, including the stomach and small intestine. Consequently, the in vitro digestion process concludes at the end of the intestinal phase. The intestinal phase involves the addition of simulated intestinal fluid (SIF), bile salts, digestive enzymes (pancreatin), and the establishment of a neutral pH environment. In the INFOGEST protocol, researchers may opt to utilize pancreatin, a standardized enzyme cocktail, or individual purified enzymes to simulate the intestinal phase of digestion.

**FIGURE 5:**
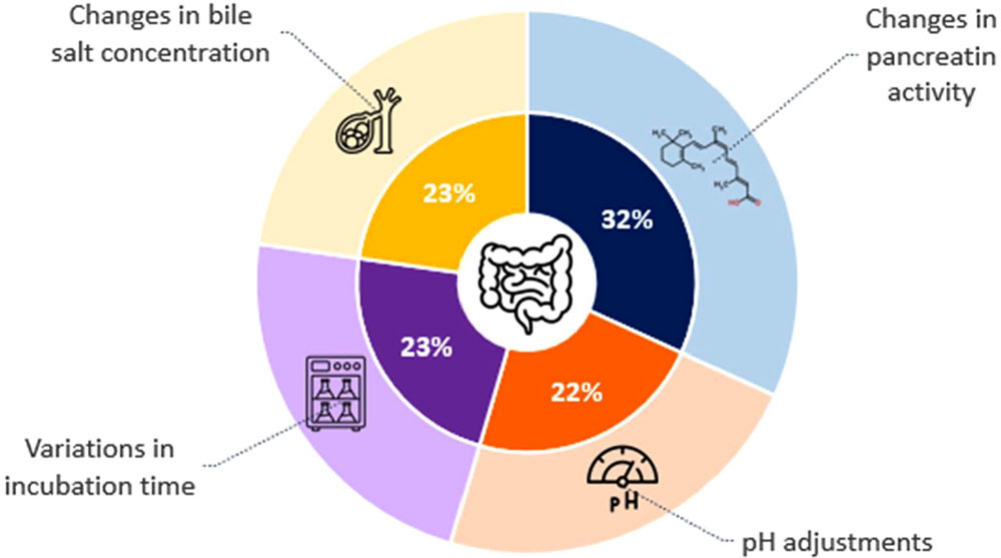
Distribution of methodological modifications reported in the intestinal phase (*n* = 23).

The main modifications identified in the intestinal phase included alterations in pancreatin activity (*n* = 7), pH adjustments (*n* = 5), variations in incubation time (*n* = 5), and changes in bile salt concentration (*n* = 5), as shown in Figure 5 and presented in Supporting Information Tables I–IV. A commonly observed modification is the alteration of pancreatin activity, specifically the trypsin activity within the pancreatin mixture. Some authors have adjusted the trypsin activity, with values ranging from 10 to 67 U/mL (Adiamo et al. 2023; Melchior et al. 2023; Moretton et al. 2023; Song et al. 2024; Wang et al. 2023; Wang, Rousta, et al. 2024), representing a decrease compared to the recommended protocol value.

These alterations were often made to adapt the in vitro digestion model to better reflect the reduced gastrointestinal enzymatic activity typically observed in elderly individuals (Adiamo et al. 2023; Melchior et al. 2023; Ménard, Lesmes, et al. 2023; Song et al. 2024). Similarly, modifications in pH values were reported, with variations between pH 6.5 and 7.5 (De Paiva Gouvêa et al. 2024; Gutiérrez‐Luna et al. 2023; Melchior et al. 2023; Moretton et al. 2023; Wang, Chang, et al. 2024). Although pH adjustments to simulate elderly digestive conditions were noted, they occurred in fewer studies (Melchior et al. 2023; Moretton et al. 2023).

Variations in incubation time were also observed, with some authors extending the duration of the intestinal phase to between 3 and 4 h (Duijsens et al. 2022; Melchior et al. 2023; Moretton et al. 2023; Noel et al. 2024). This adjustment aimed to mimic the slower intestinal transit time commonly found in older adults (Melchior et al. 2023; Moretton et al. 2023). In addition, the concentration of bile salts was often reduced in comparison to the protocol's standard of 10 mM. In several studies, bile salts levels ranged from 1 to 8.7 mM (Adiamo et al. 2023; Melchior et al. 2023; Moretton et al. 2023; Santos‐Hernández et al. 2020; Wang, Rousta, et al. 2024; Wang, Zheng, et al. 2024), with some reductions made intentionally to simulate the age‐related decline in bile production and secretion (Melchior et al. 2023; Ménard, Lesmes, et al. 2023; Moretton et al. 2023). In other cases, lower bile salt concentrations were used to improve the biocompatibility of digests for intestinal cell culture assays, as high bile salt levels may be cytotoxic to epithelia cells (Santos‐Hernández et al. 2020; Wang, Rousta, et al. 2024; Wang, Zheng, et al. 2024). This approach is relevant because epithelia cells are widely used as an in vitro model of the human intestinal epithelium, helping to assess whether nutrients such as amino acids and minerals released during digestion are potentially available for cellular uptake and transport (Wang, Rousta, et al. 2024).

Collectively, these adaptations reflect an effort to increase physiological relevance for specific population groups, such as the elderly, but they may reduce the reproducibility and comparability of findings across studies strictly following the INFOGEST protocol.

### 3.2 Other Modifications

Additional modifications to the protocol were introduced to meet specific experimental needs. In two studies, the assay used to determine pancreatin enzymatic activity was modified by measuring trypsin activity in the supernatant of the pancreatin solution (Auer et al. 2024; Sousa et al. 2023a). In Muleya, Young, et al. (2023), stable isotopes were added to the simulated digestive fluids to facilitate the analysis of mineral bioaccessibility in food samples, including iron, calcium and nitrogen (^1^ ^5^N). Specifically, the simulated salivary, gastric, pancreatic, and bile fluids were labeled to serve as a tracer for reagent‐derived iron. This modification allows for the quantitative discrimination between the iron native to the food sample and the residual iron present in digestion reagents, such as pepsin, pancreatin, and bovine bile, which could otherwise lead to an overestimation of bioaccessibility (Muleya et al. 2024). Similarly, Ménard, Chauvet, et al. (2023), used intrinsically ^1^ ^5^N‐labeled gluten and caseins to discriminate dietary nitrogen from endogenous nitrogen derived mainly from pancreatin and bile. This distinction is important because endogenous nitrogen may substantially contribute to the digestible fraction and lead to an overestimation of apparent digestibility when not properly accounted for.

Additionally, in the oral phase, some authors have incorporated mucin (*n* = 5), a salivary glycoprotein naturally present in human saliva that plays key roles in lubrication, pellicle formation, and antimicrobial defense. Mucin can also interact with ingested biopolymers, thereby contributing to more physiologically realistic digestion models (Ozel et al. 2020). However, mucin is a minor component of saliva and was therefore excluded from the original INFOGEST protocol (Minekus et al. 2014).

Although many of the methodological modifications discussed throughout the digestion phases were introduced to increase physiological relevance, several studies also demonstrate that these adaptations may be driven by analytical and matrix‐related requirements rather than exclusively by the attempt to mimic human physiology.

For filamentous fungal biomass, Wang et al. (2023) reduced pancreatin (10 U/mL) and salivary amylase (7.5 U/mL) activities and excluded ammonium carbonate (NH_4_CO_3_) from the simulated digestive fluids to minimize interference from reagent‐derived primary amines during OPA‐based determination of the degree of hydrolysis. While these modifications improved analytical accuracy, the lower pancreatin activity limited protein hydrolysis in samples with higher protein contents, and the extended oral phase compensated for the reduced amylase activity.

In contrast, Guice et al. (2023) supplemented the gastric phase with inulinase during the digestion of plant‐based burgers, significantly enhancing fructan hydrolysis, as demonstrated by the increased release of free fructose compared with the standard INFOGEST protocol. Most fructan degradation occurred during the gastric phase, with no additional benefit from the intestinal phase, whereas enzyme doses ≥ 400 inulinase units (INU) also promoted greater food matrix disintegration.

Finally, Miedes et al. (2023) incorporated cholesterol esterase into the INFOGEST 2.0 protocol to better simulate phytosterol digestion and replaced in vivo mastication adapted for older adults with partially dehydrated bread to reduce variability associated with inadequate food disintegration. Furthermore, the high fiber content of whole‐grain rye bread increased digesta viscosity and reduced lipolysis and micellization, resulting in lower phytosterol bioaccessibility.

Solid and complex plant matrices often require specific preparation strategies to ensure homogeneity and reproducibility before in vitro digestion. In cereal‐ and legume‐based systems, approaches such as controlled size reduction, standardized milling, and preprocessing have been used to minimize variability related to heterogeneous particle distribution and oral processing simulation (Baugreet et al. 2019; Adiamo et al. 2023; Abdelbost et al. 2024). These adaptations indicate that, in heterogeneous plant matrices, reproducibility may depend on physical standardization of the sample before digestion. For protein‐rich materials, including germinated or minimally processed grains, pretreatment steps have also been applied to improve matrix uniformity and facilitate enzymatic access during digestion assays (Abdelbost et al. 2024; Hu et al. 2023).

Food structure and processing history may further influence digestion behavior, particularly protein and starch hydrolysis. Differences between intact and processed cereal structures can alter enzyme accessibility and digestion kinetics (Duijsens et al. 2022; Purwandari et al. 2023), while thermal processing and ingredient restructuring may affect protein denaturation and starch gelatinization, thereby modifying enzymatic hydrolysis during simulated gastrointestinal digestion (Zahir et al. 2021).

In emulsified matrices and plant‐based shakes, additional techniques such as pH‐stat monitoring (Chen et al. 2023; Infantes‐Garcia et al. 2022) and high‐pressure homogenization (Guevara‐Zambrano et al. 2022; Melchior et al. 2023) were incorporated to better characterize lipolysis kinetics and interfacial digestion phenomena associated with the structural properties of the food matrix. Likewise, dialysis membranes (Naik et al. 2024; Hu et al. 2023) and blank corrections (Auer et al. 2024; Muleya et al. 2024; Canelli et al. 2023) were applied in studies evaluating minerals and phenolic compounds to refine estimates of intestinal bioaccessibility and minimize analytical interference derived from digestion reagents.

## 4 Conclusion

This systematic review shows that methodological adaptations of the static INFOGEST 2.0 protocol are widely applied in studies investigating plant‐based foods and supplements. However, in most cases, these adaptations are not inherently linked to the plant‐based nature of the matrices. Rather, they arise from two distinct methodological drivers: adjustments introduced to address analytical and experimental requirements associated with specific study designs, and adaptations to simulate defined physiological contexts, most notably those associated with aging. Recognizing this distinction is critical to avoid oversimplified interpretations of methodological variability in plant‐based digestion studies.

Although such adaptations can enhance physiological relevance, particularly when targeting specific populations or research objectives, they inevitably introduce a trade‐off with interstudy comparability. Modifications to consensus parameters may substantially influence digestion outcomes and obscure true matrix effects when deviations are insufficiently justified or inconsistently reported. Collectively, these findings highlight the need for clearer reporting practices and consensus‐based extensions of the INFOGEST framework that balance physiological relevance with methodological consistency.

## Author Contributions

**Matheus Braga Mendes**: writing – original draft, formal analysis, data curation. **Giovana Gomes Garcia Rodrigues**: writing – original draft, formal analysis, data curation. **Maria Esther Leal da Silva Sad**: writing – review and editing, investigation, formal analysis, data curation. **Giovanna Ferreira Paiva dos Santos**: writing – original draft, formal analysis, data curation. **Juliana de Carvalho da Costa**: conceptualization, investigation, writing – review and editing, project administration, supervision.

## Conflicts of Interest

The authors declare no conflicts of interest.

## Supporting information




**Supporting Information**: jfds71356‐sup‐0001‐SuppMat.docx
