# Supplementary material for: Methodological Modifications of the INFOGEST 2.0 Protocol in Plant‐Based Food Studies: A Systematic Review
Source: J Food Sci. 2026 Aug 21;91(8):e71356. doi: 10.1111/1750-3841.71356 (PMC13495122; doi:10.1111/1750-3841.71356)
Supplement: Supplementary file 1 [file JFDS-91-0-s001.docx]

**Table I.** Summary of studies reporting one methodological modification to the INFOGEST in vitro digestion protocol.

| **Product** | **Category** | **Micro or macronutrient analyzed** | **Modifications to the INFOGEST 2.0 protocol** | **Phase with modification** | **References** |
| --- | --- | --- | --- | --- | --- |
| Pea protein isolate | Food | Proteins | Porcine salivary amylase. |  | (Paulussen et al., 2024) |
| Chickpea flour | Food | Starch and proteins | Oral phase – water only. |  | (Pälchen et al., 2022) |
| Maize flour | Food | Proteins | Oral phase – *Bacillus sp.* amylase. |  | (Muleya et al., 2023b) |
| Breakfast cereals (yellow and purple maize) co-digested with almond milk (also tested with cow’s milk) | Food | Carotenoids | Oral phase - 10 min. |  | (Camargo et al., 2024) |
| Emulsions based on sunflower, soybean, or peanut oil | Supplement | Lipids | Oral phase - water only. |  | (Guevara-Zambrano et al., 2023) |
| · Cereals (maize, finger millet, white and brown bread, rice, oats)  · Legumes (kidney beans, lentils, chickpeas, black chickpeas, garden peas, calcium-set tofu)  · Dark green vegetables (broccoli, cabbage, kale, and spinach)  · Plant-based beverages (soy, rice, oat, and almond milk)  · Dried fruits and others (raisins, prunes, figs, apricots, tahini) | Food | Calcium | Addition of stable calcium isotope to digestion fluids;  *Bacillus sp* amylase. |  | (Muleya; F. Bailey; H. Bailey, 2024) |
| Pearl millet grain (iron contamination from soil) | Food | Iron | Iron isotopic labeling (⁵⁷Fe) in digestion fluids;  *Bacillus sp* amylase. |  | (Muleya et al., 2023a) |
| Maillard reaction product (MRP) conjugate system based on amaranth and the seaweed Gracilaria | Supplement | Antioxidants and amino acids | Higher fluid-to-sample ratio (1:50). |  | (Naik et al., 2024) |
| *Galdieria sulphuraria (microalgae)* | Food | Proteins | Higher fluid-to-sample ratio (1:7). |  | (Canelli et al., 2023) |
| Soy milk fortified with curcumin, quercetin, or resveratrol | Food | Polyphenols | Mucin in the oral phase. |  | (Zheng; Zhou; McClements, 2023). |
| Isolates: pea–soy, soy (concentrate), peanut–pea | Food | Proteins | Reduced bile salts (2.5 mM). |  | (Wang et al., 2024c) |
| Garden pea, grass pea, soybean and lentil protein isolate powders | Food | Proteins | Reduced bile salts (2.5 mM). |  | (Santos-Hernández et al., 2020) |
| Plant-based yogurt | Food | All mineral elements | No gastric lipase. |  | (Rebellato et al., 2023) |
| Sorghum flour | Food | Proteins | Gastric phase - pepsin (400 U/mL), pH 2.0. |  | (Abdelbost et al., 2024) |
| Plant-based beverages (rice, almond, oat, cashew, peanut, coconut, and soy) | Food | Inorganic elements (potentially toxic) | No gastric lipase. |  | (Fioravanti et al., 2023) |
| Phenolic standards | Supplement | Phenolic compounds (flavonols, flavanones, flavan-3-ols, and phenolic acids) | No gastric lipase. |  | (Pais et al., 2024) |
| Microalga (Chlorella vulgaris) | Food | Proteins | No gastric lipase. |  | (Van De Walle et al., 2024) |
| Sunflower seed, soybean, and nuts | Food | Lipids | No gastric lipase. |  | (Huyan et al., 2024) |
| Yeast extract from wine lees (fermentation sediments) | Food | Proteins | No gastric lipase. |  | (Moreira et al., 2024) |
| Isolated cotyledon cells from jack bean (Canavalia ensiformis) | Food | Proteins and starch | No gastric lipase. |  | (Purwandari et al., 2023) |
| Soybean (germinated and non-germinated: intact cotyledon and flour) | Food | Proteins | No gastric lipase. |  | (Zahir; Fogliano; Capuano, 2021) |
| Coconut shell | Food | Phenolic compounds | No gastric lipase. |  | (Cañas et al., 2024) |
| Plant-based meat analog (pea protein) | Food | Metabolites (phenolic compounds) | No gastric lipase. |  | (Izquierdo-Sandoval et al., 2024) |
| Bigel (biphasic encapsulation system composed of an oleogel and a hydrogel phase) | Supplement | Curcumin (a polyphenol from turmeric) | Gastric phase - pH 2.0. |  | (Kurapkienė et al., 2024) |
| Potato (protein isolate in different matrices: suspension, foam, gel, and heat-set foam) | Supplement | Proteins | No gastric lipase. |  | (Jiménez-Munoz; Nielsen; Corredig, 2024) |
| Textured soy protein | Food | Proteins | No gastric lipase. |  | (Yao et al., 2024) |
| Soy protein burger | Food | Proteins | NaHCO₃ replaced with NaCl in digestion fluid;  No gastric lipase. |  | (Zhou et al., 2021) |
| Microbial biomass | Supplement | Proteins | No gastric lipase. |  | (Nordlund et al., 2024) |
| Nanoemulsions enriched with α-tocopherol (avocado, flaxseed, and chia oils) | Supplement | Lipids and α-tocopherol | Gastric phase - 1.5 h. |  | (Maldonado et al., 2023) |
| Black bean burger, brussels sprouts, garlic, and onion | Food | Fibers | Addition of the enzyme inulinase. |  | (Guice et al., 2023) |
| Lentil powder | Food | Starch and proteins | Intestinal phase - 3 h. |  | (Duijsens et al., 2022) |
| Soybean, pea, and faba bean | Food | Proteins, iron e zinc | Modified pancreatin enzyme activity assay. |  | (Auer et al., 2024) |
| Pea cell wall; pea protein plus polysaccharides; pea protein plus incubation solutions | Food | Proteins | Intestinal phase - 4 h. |  | (Noel et al., 2024) |

**Table II.** Summary of studies reporting two methodological modifications to the INFOGEST in vitro digestion protocol.

| **Product** | **Category** | **Micro or macronutrient analyzed** | **Modifications to the INFOGEST 2.0 protocol** | **Phase with modification** | **References** |
| --- | --- | --- | --- | --- | --- |
| Shake (pea protein isolate and soy lecithin) | Food | Lipids and proteins | Oral phase – water only;  Phenylmethylsulfonyl fluoride added to inhibit proteases. |  | (Guevara-Zambrano et al., 2022) |
| Pea protein isolate, rice protein, and lentil flour | Food | Proteins | Oral phase performed by the author (self-chewing model);  Gastric phase – CaCl₂ (150 mM. |  | (Baugreet et al., 2019) |
| Carrots | Food | β-Carotene | Oral phase – volunteers (20-41 seg.);  No gastric lipase. |  | (Chen; Capuano; Stieger, 2024) |
| κ-carrageenan/protein microspheres (soy and pea) | Supplement | Proteins | Mucin added in oral phase;  No gastric lipase. |  | (Ozel et al., 2020) |
| Triolein-based emulsion | Supplement | Lipids | Oral phase - water only;  No Intestinal phase. |  | (Infantes-Garcia et al., 2022) |
| Common bean | Food | Iron and zinc | Oral phase – amylase (100 U/mL);  Intestinal phase – dialysis membrane inserted. |  | (Huertas et al., 2022) |
| Soy and pea–faba bean burgers | Food | Proteins | Amylase (300 U/mL);  Modified pancreatin enzyme activity assay. |  | (Sousa et al., 2023b) |
| Soybean meal, barley, wheat, pea, faba bean, chickpea, maize, and beet pulp | Food | Proteins | No gastric lipase;   Temperature modified to 39 °C. |  | (Grundy et al., 2022) |
| Sesame protein isolate | Food | Proteins | Higher fluid-to-sample ratio (1:20);  No gastric lipase. |  | (Hu et al., 2023) |
| Pea protein isolate | Food | Proteins | Oral phase enzyme replaced with water;  No gastric lipase. |  | (Guevara-Zambrano et al., 2024) |
| Cooked lentil | Food | Proteins | Higher fluid-to-sample ratio (1:2);  No gastric lipase. |  | (Verkempinck et al., 2022) |
| Soy protein isolate and gluten | Food | Proteins | Gastric phase (elderly) - pepsin (800 U/mL);  Intestinal phase (elderly) - trypsin (50 U/mL). |  | (Song et al., 2024) |
| Scallop analogue (pea protein) | Food | Proteins | Mucin in the oral phase; Replacement of NaHCO₃ with NaCl in the digestive fluids. |  | (Zhang et al., 2023) |
| Plant-based chicken (potato protein) | Food | Proteins and lipids | Mucin in the oral phase; Replacement of NaHCO₃ with NaCl in the digestive fluids. |  | (Ryu; Zhou; McClements, 2024). |
| Lutein-fortified plant-based eggs | Food | Proteins and lipids | Mucin in the oral phase; Replacement of NaHCO₃ with NaCl in the digestive fluids. |  | (Vu et al.; 2022) |
| Wholegrain rye bread enriched with plant sterols | Food | Lipids | Human oral phase (α-amylase, 98 U/mL); Addition of the enzyme cholesterol esterase. |  | (Faubel et al., 2022) |

**Table III.** Summary of studies reporting three methodological modifications to the INFOGEST in vitro digestion protocol.

| **Product** | **Category** | **Micro or macronutrient analyzed** | **Modifications to the INFOGEST 2.0 protocol** | **Phase with modification** | **References** |
| --- | --- | --- | --- | --- | --- |
| Bread (conventional – wheat flour; enriched – pea protein concentrate) | Food | Proteins and carbohydrates | Oral phase - healthy adult and elderly volunteers (15 seg.);  Elderly gastric phase – altered enzyme concentrations, pH 4.5, 3 h;  Elderly intestinal phase – altered enzyme concentrations, bile salts (6.6 mM), pH 6.6, 3 h. |  | (Moretton et al., 2023) |
| Olive oil-based gelled emulsion | Supplement | Fatty acids and antioxidants | Oral phase - pH 6.5;  Gastric phase - pH 2.5;  Intestinal phase - pH 7.5. |  | (Gutiérrez-Luna; Astiasaran; Ansorena, 2023) |
| Filamentous fungus (Rhizopus microsporus var. oligosporus) grown on oat flour | Supplement | Proteins, amino acids, and minerals | Oral phase - amylase (7.5 U/mL), 1 h incubation;  Gastric phase - No gastric lipase;  Intestinal phase - pancreatin (10 U/mL) and bile salts (1 mM). |  | (Wang et al., 2024a) |
| Protein isolates (soy and pea); protein concentrates (soy, pea, common bean, and faba bean); common bean and faba bean flours | Food | Proteins | Higher sample proportion (2:1);  Gastric phase - pH 2.0;  Intestinal phase - pH 7.5. |  | (De Paiva Gouvêa et al., 2024) |
| *Camellia oleifera oil body* | Food | Lipids | No gastric lipase;  Gastric phase - CaCl2 (0.005 mM);  Intestinal phase - CaCl2 (0.3 mM). |  | (Chen et al., 2023) |
| Wholegrain rye bread enriched with plant sterols | Food | Lipids | Gastric phase (elderly) – 2.5 h, pH = 6.0, gastric lipase (9 U/mL), pepsin (1500 U/mL); Inclusion of cholesterol esterase in the intestinal phase (0.075 U/mL) for adults and elderly individuals; Intestinal phase (elderly) – 2.5 h, pancreatin (50 U/mL), bile salts (5 mM). |  | (Miedes et al., 2023) |

**Table IV.** Summary of studies reporting four or more methodological modifications to the INFOGEST in vitro digestion protocol.

| **Product** | **Category** | **Micro or macronutrient analyzed** | **Modifications to the INFOGEST 2.0 protocol** | **Phase with modification** | **References** |
| --- | --- | --- | --- | --- | --- |
| Lentil and chickpea powder | Food | Proteins | No gastric lipase;  Oral phase - CaCl2 (0.93 mM);  increased gastric fluid volume;  Intestinal phase – individual enzymes combined with pancreatin. |  | (Duijsens et al., 2023) |
| Wheat gluten (concentrate), pea, and rice (protein concentrate) | Food | Proteins | Porcine pancreatic amylase type VI-B;  Elderly oral phase – amylase (150 U/mL) and KCl (30.1 mmol/L);  Elderly gastric phase – pepsin (1500 U/mL), pH 5.0, 3 h;  Elderly intestinal phase – pancreatin (46 U/mL), bile salts (59.4 mg/mL), pH 6.6, 3 h. |  | (Melchior et al., 2023) |
| Filamentous fungal biomass | Supplement | Proteins and amino acids | Oral phase - amylase (7.5 U/mL), 1 h;  Gastric phase - No gastric lipase;  Intestinal phase - 10 U/mL pancreatin;  No NH₄CO₃ added to simulated digestive fluids. |  | (Wang et al., 2023) |
| Australian Acacia species protein concentrate | Food | Proteins | Higher fluid-to-sample ratio (1:10);  No gastric lipase;  Gastric phase - 1 h;  Intestinal phase - lower fluid ratio (2:1), bile salts (2 mM), CaCl₂ (0.2 mM), trypsin (67 U/mL), chymotrypsin (18 U/mL). |  | (Adiamo et al., 2023) |
| Galactomannan-epigallocatechin gallate complex | Supplement | Polysaccharides and polyphenols | Higher fluid-to-sample ratio (1:50);  Oral phase - 5 min.;  Gastric phase - pH 2.0;  Intestinal phase - pH 6.0. |  | (Wang et al., 2024b) |

**References**

Chen, Y., E. Capuano, and M. Stieger. 2024. “The effects of carrot shape and oral processing behaviour on bolus properties and β-carotene bioaccessibility of raw carrots”. *Food Research International*, *179*, 114051.

Camargo, C. A., R. Salvador-Reyes, C. S. R. Bazzani, M. T. P. S. Clerici, M. C. Marques, and L. R. B. Mariutti. 2024. “Screening the Carotenoid In Vitro Bioaccessibility of Purple Corn Breakfast Cereal Consumed With Milk and Plant-Based Milk.” *Food Research International* 197: 115259.

Duijsens, D., K. Pälchen, S. Verkempinck, et al. 2023. “Size Exclusion Chromatography to Evaluate In Vitro Proteolysis: A Case Study on the Impact of Microstructure in Pulse Powders.” *Food Chemistry* 418: 135709.

Faubel, N., M. Makran, A. Cilla, A. Alegria, R. Barbera, and G. Garcia-Llatas. 2022. “Bioaccessibility of plant sterols in wholemeal rye bread using the INFOGEST protocol: Influence of oral phase and enzymes of lipid metabolism”. *Journal of Agricultural and Food Chemistry*, *70*(41), 13223.

Fioravanti, M. I. A., A. P. Rebellato, R. F. Milani, M. A. Morgano, and A. P. A. Bragotto. 2023. “Toxic Inorganic Elements in Plant-Based Beverages: Total Concentration, Dietary Exposure and Bioaccessibility.” *Journal of Food Composition and Analysis* 123: 105565.

Grundy, M. M. L., J. Tang, J. van Milgen, and D. Renaudeau. 2022. “Cell Wall of Feeds and Their Impact on Protein Digestibility: An In Vitro Method Applied for Pig Nutrition.” *Animal Feed Science and Technology* 293: 115467.

Guevara-Zambrano, J. M., D. Michels, S. H. E. Verkempinck, et al. 2023. “HPLC-CAD Method to Quantify Lipolysis Products From Plant-Based Oils Rich in Unsaturated Fatty Acids.” *Journal of Food Composition and Analysis* 121: 105400.

Guevara-Zambrano, J. M., P. Chowdhury, A. G. B. Wouters, and S. H. E. Verkempinck. 2024. “Solubility, (Micro)Structure, and In Vitro Digestion of Pea Protein Dispersions as Affected by High Pressure Homogenization and Environmental Conditions.” *Food Research International* 188: 114434.

Huertas, R., J. William Allwood, R. D. Hancock, and D. Stewart. 2022. “Iron and Zinc Bioavailability in Common Bean ( Phaseolus vulgaris ) Is Dependent on Chemical Composition and Cooking Method.” *Food Chemistry* 387: 132900.

Huyan, Z., N. Pellegrini, J. Rubert, W. T. Steegenga, and E. Capuano. 2024. “Levels of Lipid-Derived Gut Microbial Metabolites Differ Among Plant Matrices in an In Vitro Model of Colon Fermentation.” *Food Research International* 184: 114230.

Izquierdo-Sandoval, D., X. Duan, C. Fryganas, T. Portolés, J. V. Sancho, and J. Rubert. 2024. “Untargeted Metabolomics Unravels Distinct Gut Microbial Metabolites Derived From Plant-Based and Animal-Origin Proteins Using In Vitro Modeling.” *Food Chemistry* 457: 140161.

Jiménez-Munoz, L., S. D.-H. Nielsen, and M. Corredig. 2024. “Changes in Potato Peptide Bioactivity After Gastrointestinal Digestion. An In Silico and In Vitro Study of Different Patatin-rich Potato Protein Isolate Matrices.” *Food Chemistry Advances* 4: 100679.

Nordlund, E., P. Silventoinen-Veijalainen, T. Hyytiäinen-Pabst, et al. 2024. “In Vitro Protein Digestion and Carbohydrate Colon Fermentation of Microbial Biomass Samples From Bacterial, Filamentous Fungus and Yeast Sources.” *Food Research International* 182: 114146.

Pais, A. C. S., E. R. Coscueta, M. M. Pintado, A. J. D. Silvestre, and S. A. O. Santos. 2024. “Exploring the Bioaccessibility and Intestinal Absorption of Major Classes of Pure Phenolic Compounds Using In Vitro Simulated Gastrointestinal Digestion.” *Heliyon* 10, no. 7: e28894.

Pälchen, K., W. L. P. Bredie, D. Duijsens, et al. 2022. “Effect of Processing and Microstructural Properties of Chickpea-Flours on In Vitro Digestion and Appetite Sensations.” *Food Research International* 157: 111245.

Paulussen, K. J. M., A. T. Askow, M. T. Deutz, et al. 2024. “Acute Microbial Protease Supplementation Increases Net Postprandial Plasma Amino Acid Concentrations After Pea Protein Ingestion in Healthy Adults: A Randomized, Double-Blind, Placebo-Controlled Trial.” *Journal of Nutrition* 154, no. 5: 1549–1560.

Rebellato, A. P., M. I. A. Fioravanti, R. F. Milani, and M. A. Morgano. 2023. “Composition and Bioaccessibility of Inorganic Elements in Plant-Based Yogurts.” *Journal of Food Composition and Analysis* 123: 105639.

Ryu, J., H. Zhou, and D. J. McClements. 2024. “In vitro digestibility and gastrointestinal fate of plant-based chicken analogs prepared using a soft matter physics approach”. *Food Biophysics*, *19*(1), 109-119.

Sousa, R., I. Recio, D. Heimo, et al. 2023b. “In Vitro Digestibility of Dietary Proteins and In Vitro DIAAS Analytical Workflow Based on the INFOGEST Static Protocol and Its Validation With In Vivo Data.” *Food Chemistry* 404, no. pt B: 134720.

Verkempinck, S. H. E., D. Duijsens, D. Michels, et al. 2022. “Studying Semi-Dynamic Digestion Kinetics of Food: Establishing a Computer- Controlled Multireactor Approach.” *Food Research International* 156: 111301.

Vu, G., X. Xiang, H. Zhou, and D. J. McClements. 2022. “Lutein-fortified plant-based egg analogs designed to improve eye health: formation, characterization, in vitro digestion, and bioaccessibility”. *Foods*, *12*(1), 2.

Yao, Y., Y. Zheng, Y. I. Zhang, and H. Zeng. 2024. “Pressure-Dominated Steam Explosion for Modifying Textured Soy Proteins: Structure and In Vitro Digestion Kinetics.” *Food Research International* 180: 114071.

Zhang, Z., D. Qin, K. Kobata, J. Rao, J. Lu, and D. J. McClements. 2023. “An in vitro comparison of the digestibility and gastrointestinal fate of scallops and plant-based scallop analogs”. *Foods*, *12*(15), 2928.

Zheng, B., H. Zhou, and D. J. McClements. 2023. “Co-encapsulation of multiple polyphenols in plant-based milks: Formulation, gastrointestinal stability, and bioaccessibility”. *Foods*, *12*(18), 3432.

Zhou, H., Y. Hu, Y. Tan, Z. Zhang, and D. J. McClements. 2021. “Digestibility and Gastrointestinal Fate of Meat Versus Plant-Based Meat Analogs: An In Vitro Comparison.” *Food Chemistry* 364: 130439.
